# MgrB-Dependent Colistin Resistance in Klebsiella pneumoniae Is Associated with an Increase in Host-to-Host Transmission

**DOI:** 10.1128/mbio.03595-21

**Published:** 2022-03-21

**Authors:** Andrew S. Bray, Richard D. Smith, Andrew W. Hudson, Giovanna E. Hernandez, Taylor M. Young, Hannah E. George, Robert K. Ernst, M. Ammar Zafar

**Affiliations:** a Department of Microbiology and Immunology, Wake Forest School of Medicine, Winston Salem, North Carolina, USA; b Department of Microbial Pathogenesis, University of Maryland, Baltimore, Baltimore, Maryland, USA; c Department of Pathology, University of Maryland, Baltimore, Baltimore, Maryland, USA; d Wake Forest University, Winston Salem, North Carolina, USA; University of Michigan Medical School; Fred Hutchinson Cancer Research Center

**Keywords:** antimicrobial peptides, gastrointestinal infection, host-to-host transmission, infection control, stress adaptation, two-component regulatory systems

## Abstract

Due to its high transmissibility, Klebsiella pneumoniae is one of the leading causes of nosocomial infections. Here, we studied the biological cost of colistin resistance, an antibiotic of last resort, in this opportunistic pathogen using a murine model of gut colonization and transmission. Colistin resistance in K. pneumoniae is commonly the result of the inactivation of the small regulatory protein MgrB. Without a functional MgrB, the two-component system PhoPQ is constitutively active, leading to an increase in lipid A modifications and subsequent colistin resistance. Using an isogenic *mgrB* deletion mutant (MgrB^−^), we demonstrate that the mutant’s colistin resistance is not associated with a fitness defect under *in vitro* growth conditions. However, in our murine model of K. pneumoniae gastrointestinal (GI) colonization, the MgrB^−^ colonizes the gut poorly, allowing us to identify a fitness cost. Moreover, the MgrB^−^ mutant has higher survival outside the host compared with the parental strain. We attribute this enhanced survivability to dysregulation of the PhoPQ two-component system and accumulation of the master stress regulator RpoS. The enhanced survival of MgrB^−^ may be critical for its rapid host-to-host transmission observed in our model. Together, our data using multiple clinical isolates demonstrate that MgrB-dependent colistin resistance in K. pneumoniae comes with a biological cost in gut colonization. However, this cost is mitigated by enhanced survival outside the host and consequently increases its host-to-host transmission. Additionally, it underscores the importance of considering the entire life cycle of a pathogen to determine the actual biological cost associated with antibiotic resistance.

## INTRODUCTION

Host-to-host transmission of antimicrobial-resistant bacteria resulting in nosocomial infections is a significant public health concern and associated with hundreds of billions of dollars in health care costs (1, 2). At the forefront of this threat is the Gram-negative pathogen Klebsiella pneumoniae, which is responsible for the second-highest frequency of hospital-acquired infections in Europe (3). Carbapenem-resistant and extended-spectrum β-lactam-resistant K. pneumoniae are associated with poor patient outcomes (4, 5). The rise in infections due to multidrug-resistant (MDR) K. pneumoniae has resulted in a correlative increase in the use of last-resort antibiotics, such as polymyxins (6, 7). Polymyxins are cationic antimicrobial peptides (CAMPs) that disrupt the outer membranes of Gram-negative bacteria by interacting with the negatively charged lipopolysaccharides (LPSs) on the bacterial membrane surface (8). Unfortunately, the increasing use of polymyxins has fueled the generation of polymyxin-resistant K. pneumoniae strains (9). While initially reported outbreaks of MDR and polymyxin-resistant K. pneumoniae were largely in Southeast Asia, these strains have now spread around the globe (10–13).

Clinically, there are two commonly used polymyxins, namely, colistin (polymyxin E) and polymyxin B, which share a similar structure of a cationic peptide ring with a linear chain of three amino acids that connect to a hydrophobic acyl tail (6, 8). Treatment with colistin is considered a salvage therapy for patients infected with MDR Gram-negative bacteria (6, 7, 14). Colistin resistance is well characterized in K. pneumoniae and involves modifications to the negatively charged lipid A moiety of the lipopolysaccharide (LPS) (15) that occur through mutations resulting in dysregulation of the two-component systems (TCSs) PmrAB, PhoPQ, or CrrAB or acquisition of a plasmid with the mobilized colistin resistance gene (*mcr1*) (16–19).

Despite the contribution of the multiple TCSs in lipid A modification, colistin resistance in the majority (∼70%) of K. pneumoniae strains is caused by mutations that abrogate the function of the small regulatory protein MgrB (20–22). MgrB localizes to the inner membrane and negatively regulates PhoPQ activity. Consequently, PhoPQ is overactivated when MgrB is inactivated, resulting in an increase in the lipid A modifications that confer colistin resistance (17, 23). The PhoPQ TCS is critical for colistin resistance in K. pneumoniae because PhoP not only directly upregulates genes that are responsible for palmitoylation, hydroxylation, and glycosylation (*pagP*, *lpxO*, and the *arn* operon) of the lipid A but also upregulates PmrA which drives glycosylation (*arn* operon and *ugd*) and the addition of phosphoethanolamine (*pmrC*) (17, 24–28).

Antimicrobial resistance is associated with specific biological functions of the cell, and consequently, it is generally associated with a biological cost. The biological cost of antibiotic resistance can manifest as altered bacterial growth, virulence, or transmissibility (29). The cost of colistin resistance has been studied in a variety of *Enterobacteriaceae* members, including K. pneumoniae, with conflicting results (30–33). The inconsistent data on biological cost can be attributed to the type of mutation, the strain tested, and the type of assays conducted (*in vivo* and *in vitro*) (30–33). However, studies investigating the biological cost of colistin resistance in K. pneumoniae have neglected the potential impact on the common initial colonization step—the gastrointestinal tract (GI)—as well as host-to-host transmission (32, 33). Epidemiological data suggest the GI tract to be the initial site of colonization for K. pneumoniae, from where it can cause disease in the same individual or transmit to another host (34). Our recent murine model of K. pneumoniae GI colonization with an intact microbiome confirmed experimentally that K. pneumoniae colonizes the GI tract and transmits to another host through the fecal-oral route (35). Furthermore, in our murine model, we observed that the K. pneumoniae MDR isolate (KPNIH1) poorly colonized the murine GI tract compared with non-MDR isolates, suggesting a possible biological cost associated with antibiotic-resistance, although the KPNIH1 strain is genetically distinct in multiple ways from the KPPR1S strain used in this publication (35). The importance of understanding the initial GI colonization by K. pneumoniae is 2-fold. First, K. pneumoniae gut colonization is associated with poor patient outcome (34, 36). Second, K. pneumoniae is generally considered a silent gut colonizer and thus transmits asymptomatically, posing a major threat in hospital settings where multiple potential reservoirs could be silently transmitting and making it difficult to identify and control the spread of infection (35, 37).

Given the increased frequency of resistance to colistin, an antibiotic of last resort, we assessed the potential biological cost of MgrB-dependent colistin resistance by using our murine model that allows us to follow the entire life cycle of K. pneumoniae. Ultimately, this may aid in devising better strategies to combat the spread of pathogenic microbes in a hospital setting.

## RESULTS

### MgrB-dependent colistin resistance of K. pneumoniae does not affect *in vitro* growth but provides enhanced survival against CAMPs.

We first examined the biological cost of MgrB-dependent colistin resistance through growth assays using nutrient-rich media (lysogeny broth [LB]) and minimal media (M63). We compared KPPR1S (a derivative of ATCC 43816; wild type [WT]) (AZ55, see Table S1 in the supplemental material) to its isogenic mutant *ΔmgrB* (MgrB^−^) (AZ132, Table S1) and the chromosomally complemented (reconstituted at the native site) strain (MgrB^+^) (AZ141, Table S1). All three strains grew equally well with no differences in generation time under both media conditions, suggesting the MgrB^−^ strain has no fitness defect under *in vitro* growth conditions (see Fig. S1A to D in the supplemental material). To test if there is any competitive defect, we also compared the growth rates of the WT and MgrB^−^ strains cocultured in minimal media and did not observe any competitive defect *in vitro* (Fig. S1E).

10.1128/mbio.03595-21.1 FIG S1 (A and B) Growth curves (A) and generation times (B) in nutrient-rich LB media. No significant difference found using Kruskal-Wallis tests with Dunn’s test of multiple comparisons. (C and D) Growth curves (C) and generation times (D) in M63 minimal media supplemented with 0.5% fucose, an alternative carbon source present in the GI tract that can be metabolized by K. pneumoniae. No significant difference was found using Mann-Whitney *U* tests. (E) Shows competitive index (CI) of cocultured WT and MgrB^−^ strains grown in M63 minimal media supplemented with 0.5% fucose. No significant difference found using Wilcoxon signed-rank test. (A to D) Growth and generation times were done in three independent assays (in duplicate). (E) Was done in two independent assays in duplicate. (F) Polymyxin B killing assay was performed as described in Materials and Methods. Shown is the mean and SEM of three independent assays (in duplicate). Statistical differences were calculated using Kruskal-Wallis with Dunn’s test of multiple comparisons within each concentration group. **, *P < *0.01; ***, *P < *0.001; ****, *P < *0.0001. Download FIG S1, PDF file, 0.06 MB. Copyright © 2022 Bray et al. 2022 Bray et al. https://creativecommons.org/licenses/by/4.0/ This content is distributed under the terms of the Creative Commons Attribution 4.0 International license.

10.1128/mbio.03595-21.6 TABLE S1 Strains used in the study with their genotype listed. Download Table S1, DOCX file, 0.03 MB. Copyright © 2022 Bray et al. 2022 Bray et al. https://creativecommons.org/licenses/by/4.0/ This content is distributed under the terms of the Creative Commons Attribution 4.0 International license.

**FIG 1:**
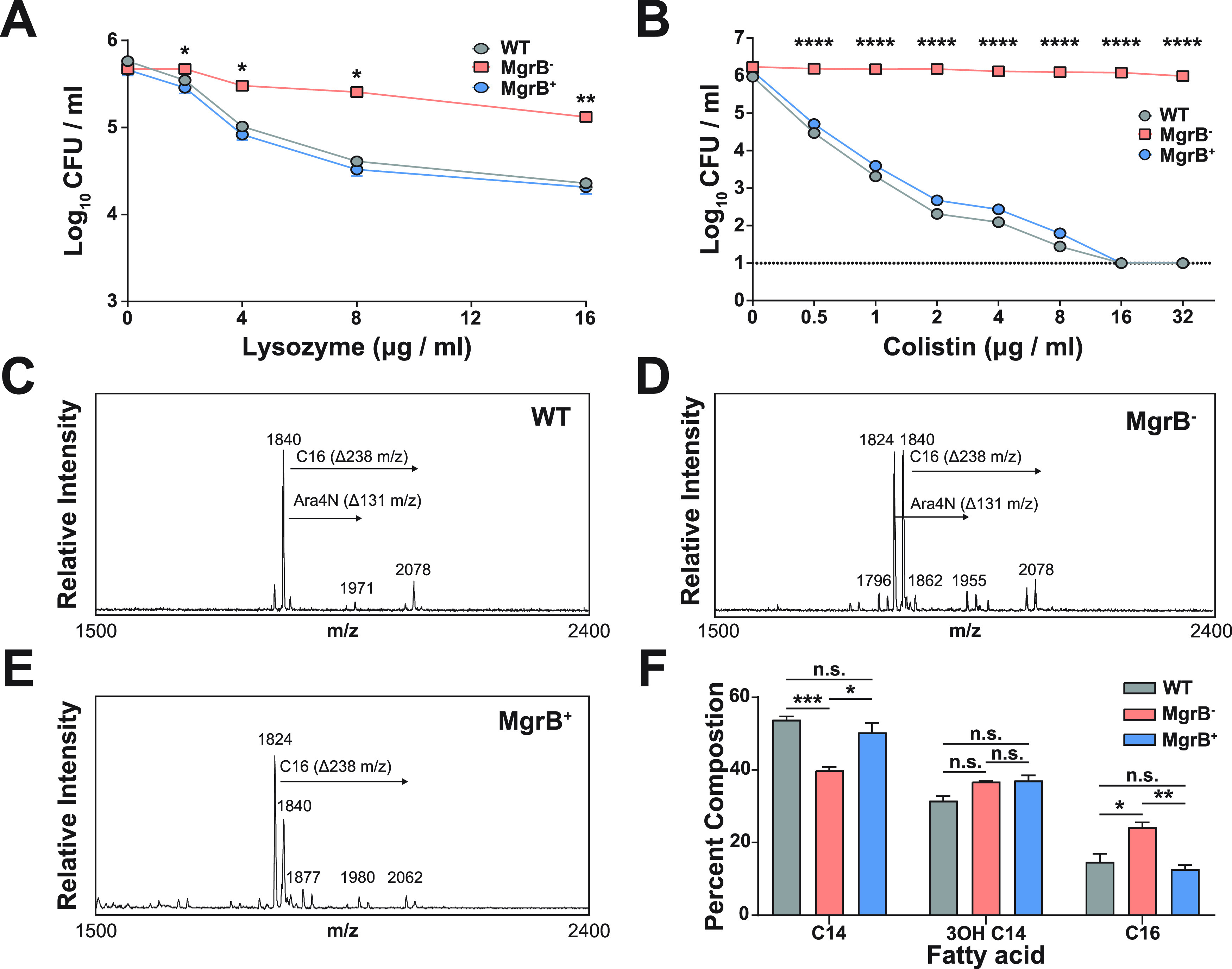
MgrB-dependent colistin resistance provides enhanced survival against CAMP. K. pneumoniae survival against lysozyme (A) and colistin (B). (A) Mid-log-phase (OD_600_) cultures of WT, MgrB^+^, and MgrB^−^ were resuspended in PBS and incubated with the appropriate concentrations of lysozyme for 1 hour at 37°C. Shown is the mean and SEM of three independent assays (in duplicate). (B) Mid-log-phase (OD_600_) cultures of each strain were diluted to ∼10^5^ CFU/mL in LB before being incubated with increasing concentrations of colistin for 30 minutes at 37°C. Shown is the mean and SEM of three independent assays (in duplicate). (C to E) Fatty acid composition was conducted using GC-FID via the fatty acid methyl ester (FAME) derivatization from a bacterial culture pellet. Strains were analyzed for lipid A analysis via FLAT with 3 biological replicates for WT (C), MgrB^−^ (D), and MgrB+ (E). (F) Fatty acid composition of lipid A of each strain. Statistical differences were calculated using Kruskal-Wallis tests with Dunn’s test of multiple comparisons within each concentration group (A and B) or fatty acid group (F). There was no significant difference between the WT and MgrB^+^ strains. *, *P < *0.05; **, *P < *0.01; ***, *P < *0.001; ****, *P < *0.0001; n.s., not significant.

The abrogated function of MgrB results in modifications of the lipid A domain of LPSs, allowing for protection against the cation-mediated targeting and disruption of the bacterial cell membrane by colistin (20, 21). In order to determine the potential impact of MgrB-dependent colistin resistance on survivability against other CAMPs we tested the survival of the WT, MgrB^−^, and MgrB^+^ strains against lysozyme, as it is one of the most abundant enzymes in the mucosal surface and has CAMP activity (38). Additionally, resistance of our MgrB^−^ strain to colistin was confirmed in a colistin (polymyxin E) and polymyxin B killing assay. MgrB^−^ had higher survival rates than the parental WT strain when treated with increasing concentrations of either lysozyme or polymyxin B/E (Fig. 1A and B) (Fig. S1F). To rule out polarity and secondary site mutations as the possible causes of the MgrB^−^ phenotype, we chromosomally complemented the strain (MgrB^+^). The MgrB^+^ strain behaved similarly to the WT, suggesting that the enhanced survival of the MgrB^−^ strain is specific to inactivation of MgrB. Taken together, our data show that MgrB inactivation provides CAMP resistance and is not associated with reduced growth *in vitro*.

Lipid A analysis for WT, MgrB^−^, and MgrB^+^ strains was analyzed via matrix-assisted laser desorption ionization–time of flight mass spectrometry (MALDI-TOF MS) after fast lipid analysis technique (FLAT) extraction (Fig. 1C to E). The fatty acid composition of the lipid A for WT, MgrB^−^, and MgrB^+^ was evaluated using gas chromatography-flame ion detection (GC-FID). As shown in Fig. 1F, the lipid A for all three strains was composed of C_14_, 3OH-C_14_, and C_16_ acyl groups. WT and MgrB^+^ lipid A had similar fatty acid composition, whereas MgrB^−^ had a significantly lower proportion of C_14_ and a higher proportion of C_16_. The difference in C_14_ and C_16_ concentrations may be explained by a potential increase in PagP activity, which adds the C_16_ palmitoyl modification to lipid A, which is upregulated by PhoP (24).

### MgrB^−^ K. pneumoniae has a fitness defect in gut colonization that is rescued transiently by antibiotic treatment.

**FIG 2:**
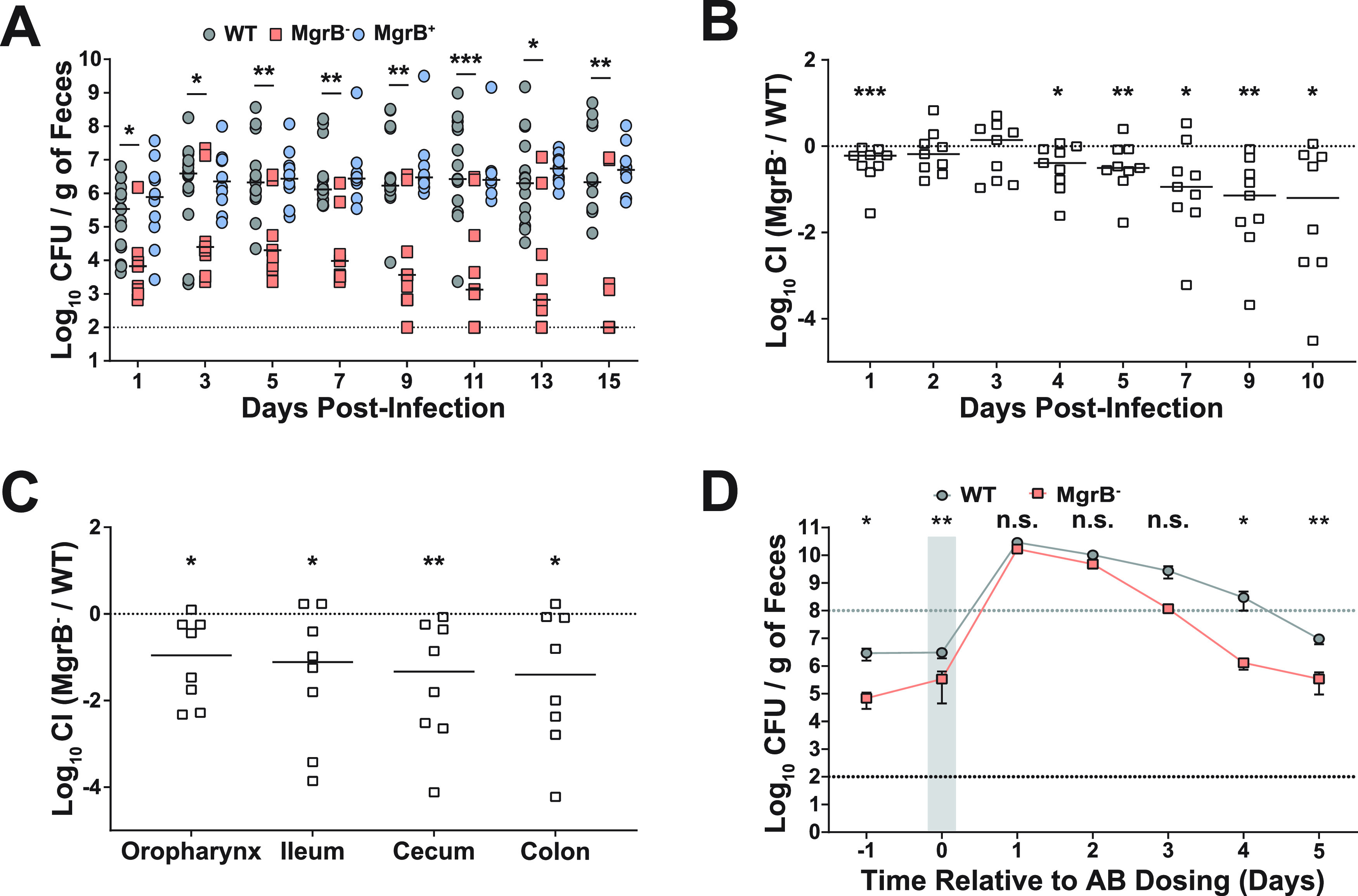
MgrB^−^ has a fitness defect in gut colonization that is rescued by antibiotic treatment. (A) Fecal shedding of infected mice. Mice were infected orally with either WT, MgrB^−^, or MgrB^+^ K. pneumoniae, and feces was collected on the days indicated (*n* ≥ 10 for each group). Each point indicates a single mouse on a given day, the bars indicate the median shedding, and the dotted line indicates the limit of detection; significance symbols shown are between WT and MgrB^−^. Kruskal-Wallis test followed by Dunn’s test of multiple comparisons was performed at each time point for analysis; WT and MgrB^+^ were not significantly different. (B) Competitive index (CI) of infected mice. Mice were infected orally with a 1:1 mixture of the WT and MgrB^−^ mutant, with feces collected on the days indicated (*n* ≥ 10). The CI was determined as described in Materials and Methods. Each symbol represents the log_10_ CI value from an individual mouse on a given day, with a bar indicating the median value. The dashed line indicates a competitive index of 1 or a 1:1 ratio of WT to mutant. (C) Colonization density of the colon, cecum, ileum, and oropharynx represented as log_10_ CI values from an individual mouse at the end of the study. For both B and C, statistical differences were determined by Wilcoxon signed-rank test. (D) K. pneumoniae fecal shedding from mice treated with antibiotic (AB). Mice were infected orally with either WT or MgrB^−^, and 2 days postinfection, they were given a dose of 5 mg of streptomycin via oral gavage. The shaded gray area indicates the duration of antibiotic treatment. Shown are the means and standard error of the means for both WT (*n *= 6) and MgrB^−^ (*n *= 6) infected mice, the black dotted line indicates the limit of detection, and the gray dotted line indicates the super shedder threshold. Statistical differences were calculated using a Mann-Whitney *U* test at each time point. *, *P < *0.05; **, *P < *0.01; ***, *P < *0.001.

As antibiotic resistance can be associated with biological costs at various stages of a pathogen’s life cycle (colonization, disease manifestation, transmission), we decided to use our published animal model to test the ability of the MgrB^−^ strain to colonize adult, immunocompetent, non-antibiotic-treated mice (35). We inoculated mice with approximately the same dose (∼10^6^ CFU) of either WT, MgrB^−^, or MgrB^+^ strains of K. pneumoniae and enumerated bacterial shedding from their feces. Over the course of 15 days of infection, the MgrB^−^ strain shed poorly in comparison to the parental WT strain (Fig. 2A), suggesting that a potential biological cost of MgrB-dependent colistin resistance in K. pneumoniae manifests as a defect in gut colonization. The MgrB^+^ strain colonized as well as WT, indicating that the colonization defect is specific to the inactivation of MgrB. To ensure that the observed colonization defect of MgrB^−^ is not strain specific, we infected mice with a clinical fecal isolate and its isogenic *mgrB* mutant (sequence type 1322 [ST1322] and ST1322 [MgrB^−^], AZ99 and AZ179) (Table S1). We had previously shown that ST1322 colonizes the murine GI tract, albeit at a lower density (35). Compared with ST1322, the ST1322 (MgrB^−^) colonizes poorly and is cleared by the host 3 days postinfection, suggesting that the poor colonizing ability of the MgrB^−^ is not strain specific (see Fig. S2 in the supplemental material).

10.1128/mbio.03595-21.2 FIG S2 GI colonization defect due to MgrB inactivation is conserved across strains. Mice were infected orally with either the ST1322 (fecal isolate) or the *ΔmgrB* isogenic mutant (MgrB^−^), and feces was collected on the days indicated. Each point indicates a single mouse on a given day, the bars indicate the median shedding, and the dotted line indicates the limit of detection. Significance shown is between WT and MgrB^−^. Statistical significance was determined with Mann-Whitney *U* tests at each time point. *, *P < *0.05; **, *P < *0.01; n.s., not significant. Download FIG S2, PDF file, 0.1 MB. Copyright © 2022 Bray et al. 2022 Bray et al. https://creativecommons.org/licenses/by/4.0/ This content is distributed under the terms of the Creative Commons Attribution 4.0 International license.

We next assessed whether WT K. pneumoniae could compensate in *trans* for the observed defect in GI colonization by MgrB^−^ during coinfection. Coinfected mice still displayed poor shedding and colonization of MgrB^−^ K. pneumoniae compared with those of the WT (Fig. 2B and C). These observations suggest that the parental WT strain is unable to rescue the gut colonization defect of the MgrB^−^ strain. A single dose of antibiotics causes the development of a supershedder state (>10^8^ CFU/g of feces), where the amount of K. pneumoniae shed from antibiotic treated mice is ∼100-fold higher than that of mock-treated mice (35). We hypothesized that reducing the gut flora through antibiotic treatment would allow MgrB^−^ K. pneumoniae to colonize better than in the presence of normal gut flora. Mice infected with either the WT or MgrB^−^ were given a single dose of streptomycin 5 days postinfection. We found that antibiotic treatment triggered a temporary supershedder phenotype in both the WT and the MgrB^−^ strains, with both reaching the same high density in the feces (Fig. 2D). However, post-treatment, the WT strain continued to shed at a higher level longer than the MgrB^−^ strain, suggesting that the continuous treatment of antibiotics is required to bypass the intrinsic colonization defect of the MgrB^−^ strain. Together, these results show that there is a biological cost associated with inactivation of MgrB in K. pneumoniae in the context of GI colonization and that antibiotic treatment temporarily alleviates this fitness defect.

### Loss of functional MgrB impacts K. pneumoniae capsular polysaccharide production and its interaction with mucus.

**FIG 3:**
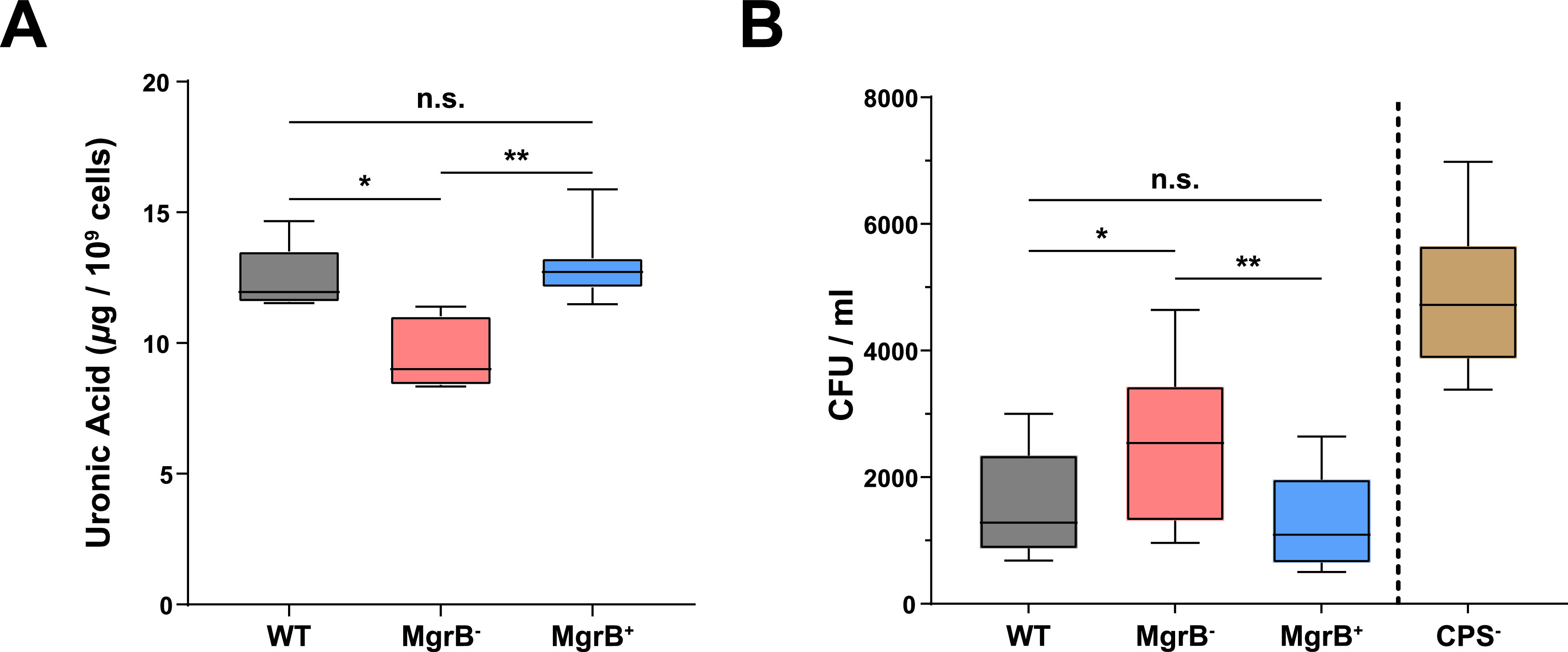
MgrB inactivation manifests as reduced capsule amount and an increased mucin association. (A) Comparison of capsule production between WT, MgrB^−^, and MgrB^+^ using a uronic acid assay from stationary-phase culture samples. (B) Comparison of the association of WT, MgrB^−^, MgrB^+^, and CPS^−^ (*Δwcaj*) to immobilized semipurified bovine submaxillary gland mucin. Shown are the CFU counts of each strain that adhered to the immobilized mucin. Boxes and whiskers indicate the means and minimum to maximum values, respectively. Three independent assays were carried out for both capsule amount (in duplicate) and mucin binding (in sextuplicate). Statistical differences were calculated using Kruskal-Wallis tests with Dunn’s test of multiple comparisons across strains. *, *P < *0.05; **, *P < *0.01; n.s., not significant.

Choi *et al.* observed variability in the production of biofilm, capsular polysaccharide (CPS), and hypermucoviscosity (HMV) between spontaneously generated colistin-resistant mutants of K. pneumoniae and their respective parental strains (33). Therefore, we assessed whether MgrB inactivation would have pleiotropic effects impacting these three K. pneumoniae virulence phenotypes. No differences were observed in biofilm formation or the HMV phenotype between the WT and MgrB^−^ strains (see Fig. S3A and B in the supplemental material). To assess whether the WT and mutant differed in the level of CPS, we quantified the amount of uronic acid, the major component of K. pneumoniae CPS produced by these strains. Compared with the WT strain, MgrB^−^ produced less CPS (Fig. 3A). We have shown previously that CPS is required for robust K. pneumoniae GI colonization (35). Additionally, CPS prevents clearance by allowing bacteria to escape the mucus present on the mucosal epithelial layer (39). Thus, we hypothesized that reduced CPS in the MgrB^−^ strain leads to entrapment in the mucus layer, eventual clearance, and thus reduced GI colonization. To determine whether a reduced capsule amount leads to increased clearance, we carried out an *in vitro* solid-phase mucin binding assay with semipurified mucin, the principal component of mucus. The unencapsulated mutant (CPS^−^, *ΔwcaJ*, AZ124) (Table S1) showed significantly higher binding to bovine submaxillary mucin than the WT strain (Fig. 3B). Moreover, the MgrB^−^ strain with reduced CPS had enhanced binding to mucin compared with the WT and MgrB^+^ strains (Fig. 3B). Taken together, our results suggest a correlation between a reduced ability to bind mucin through the production of CPS and robust GI colonization.Our findings suggest that the reduced CPS amount by the MgrB- potentially contributes towards its clearance from the GI tract.

### MgrB inactivation increases K. pneumoniae survival outside the host.

**FIG 4:**
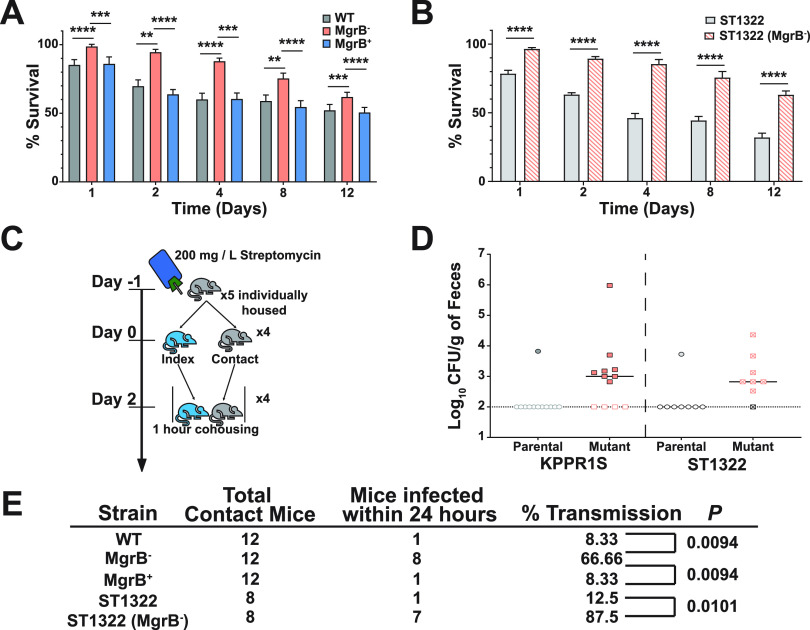
MgrB inactivation affects survival outside the host and enhances host-to-host transmission. (A and B) *Ex vivo* solid surface survival of K. pneumoniae isolate KPPR1S and ST1322, their *ΔmgrB* isogenic mutants, and the chromosomal complement *mgrB^+^*. Bacterial strains were grown overnight and resuspended in PBS, their OD_600_ was adjusted to 4, and they were spotted onto nitrocellulose discs on 1% agarose pads in 6-well polystyrene plates. Discs were removed at appropriate time intervals, bacteria were resuspended in PBS, and viable bacterial counts were determined. Shown is the mean and SEM of six independent assays (in duplicate). Statistical analysis was carried out using Kruskal-Wallis tests with Dunn’s test of multiple comparisons at each time point (A) or using Mann-Whitney *U* tests at each time point (B). **, *P < *0.01; ***, *P < *0.001; ****, *P < *0.0001. (C) Schematic representation of the protocol for the transmission studies. Mice were placed on antibiotic water 24 hours before separating and infecting the index mouse and remained on antibiotic water for the duration of the study. Once the index mouse was robustly colonized, the individually housed contact mice were exposed to the index mouse for 1 hour. Fecal shedding was collected the following day to determine if transmission had occurred. (D) Shows fecal shedding of K. pneumoniae parental isolates (KPPR1S and ST1322) and their isogenic mutant (*ΔmgrB*) from contact mice after a single exposure; the bar indicates the median shedding, and the dotted line indicates limit of detection. (E) Summary of transmission data for the indicated strains after a single 1-hour exposure. For WT, MgrB^−^, and MgrB^+^, three groups of five mice (one index and four contact) were used, and for the ST1322 strains, two groups of five mice (one index and four contact) were used. The *P* value was calculated using Fisher’s exact test.

In addition to protecting bacteria from host-mediated clearance mechanisms, the capsule contributes toward environmental survival (40). Fomites, environmental reservoirs of infections, such as catheters, ventilators, or bed handrails, are considered a significant source of K. pneumoniae transmission in a hospital setting (41), and coupled with our finding that MgrB^−^ has reduced capsule compared to the WT strain (Fig. 3A), we decided to test the environmental survivability of our strains. To determine survivability, we conducted solid surface starvation survival experiments by drying WT, MgrB^−^, and MgrB^+^ strains onto nitrocellulose membranes and placing them on agarose pads. K. pneumoniae is unable to metabolize purified agarose and nitrocellulose as energy sources, and the agarose pads allowed us to provide stable temperature and humidity. Bacteria suspended in PBS were spotted onto the agarose pads, with viable count determined over a period of 12 days. CFUs for the WT and MgrB^+^ declined rapidly over the first few time points (Fig. 4A). Unexpectedly, MgrB^−^ did not have a sharp decline in viable count and showed a significantly higher survival than the WT and the complemented strain (Fig. 4A). This finding seems contradictory as capsule is often considered a major determinant in environmental survival; however, the increased relative abundance of C_16_ observed in the LPS (Fig. 1F) has been shown to alter membrane rigidity which would potentially contribute toward increase environmental survival and serve as an alternative mechanism for colistin resistance (42). Furthermore, we tested the survivability of ST1322 and its isogenic MgrB^−^ mutant. Remarkably, the phenotype was conserved, with ST1322 (MgrB^−^) surviving at a significantly higher rate than its parental strain, ST1322 (Fig. 4B).

As fomites are a significant source of transmission in a hospital setting (41), we hypothesized that the augmented environmental survival of MgrB^−^ could manifest as enhanced host-to-host transmission. Therefore, we assessed the transmission efficiency of the MgrB^−^ and ST1322 (MgrB^−^) strains compared with that of the WT and ST1322 strains, respectively. As MgrB^−^ colonizes the GI tract poorly but reaches similar levels of colonization to WT under antibiotic pressure (Fig. 2D), mice were housed individually and given water containing streptomycin for the duration of the experiment. One mouse (index) was infected with the WT, MgrB^−^, ST1322, or ST1322 (MgrB^−^) strain, and the uninfected mice (contact) were exposed subsequently to the index mouse in the infected mouse’s cage for 1 hour each day for up to 5 days (Fig. 4C). Transmission was assessed by determining if contact mice fecal pellets were positive for K. pneumoniae (shedding). All strains colonized index mice at high density (see Fig. S4A in the supplemental material), with the MgrB^−^ index mice colonized at a slightly reduced level. After a single 1-hour exposure, more than 60% of the MgrB^−^ contact mice were colonized with MgrB^−^ K. pneumoniae, whereas less than 10% of the WT contact mice were colonized (Fig. 4D and E). Similarly, we observed higher transmission after a 1-hour exposure with the ST1322 (MgrB^−^) than that of its parental strain (80% versus 12.5%) (Fig. 4D and E). Thus, increased environmental survival correlates with higher transmission efficiency, which is not strain specific. Furthermore, once colonized, the contact mice remained colonized for the duration of the study (Fig. S4B and C).

10.1128/mbio.03595-21.3 FIG S3 MgrB inactivation does not impact biofilm formation (A) or hypermucoviscosity (B). Biofilm was calculated from 3 independent assays (in sextuplicate), and hypermucovistosity was calculated from 3 independent assays (in triplicate). There was no significant difference between strains in either assay as calculated by Kruskal-Wallis with Dunn’s test of multiple comparisons. Download FIG S3, PDF file, 0.03 MB. Copyright © 2022 Bray et al. 2022 Bray et al. https://creativecommons.org/licenses/by/4.0/ This content is distributed under the terms of the Creative Commons Attribution 4.0 International license.

10.1128/mbio.03595-21.4 FIG S4 Shedding of K. pneumoniae from index (A) and contact (B and C) mice cohoused for an hour each day with the index mouse. (B) Individual mice are color coded within each strain tested for clarity of colonization kinetics (one group of WT exposed mice was followed only for three exposures at which point all of the contact mice had detectable shedding and thus were considered to be stably colonized and were not followed further). Bars indicate median shedding, and the dotted horizontal line indicates the limit of detection. Download FIG S4, PDF file, 0.06 MB. Copyright © 2022 Bray et al. 2022 Bray et al. https://creativecommons.org/licenses/by/4.0/ This content is distributed under the terms of the Creative Commons Attribution 4.0 International license.

### Mechanism for increased environmental survival.

**FIG 5:**
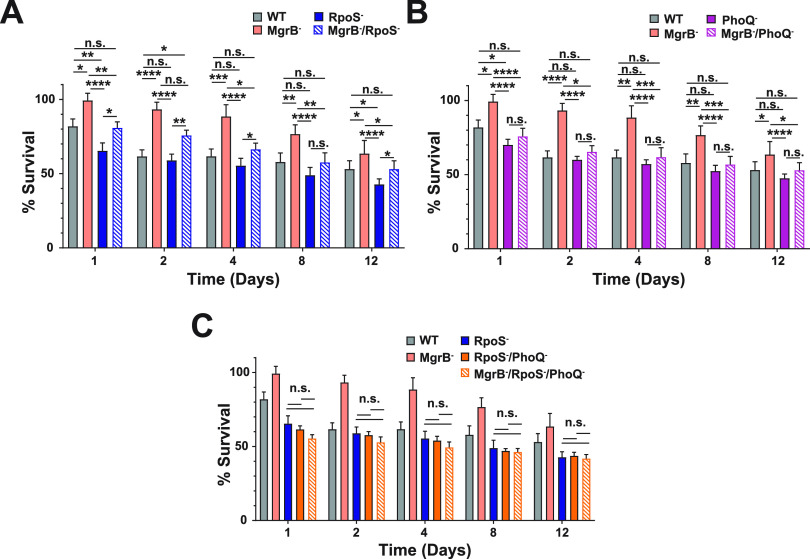
Contribution of the TCS PhoPQ and the master stress regulator RpoS to the environmental survival of K. pneumoniae. *Ex vivo* solid surface survival of KPPR1S and its isogenic mutants. (A) The impact of loss-of-function mutations in MgrB^−^, RpoS^−^, and the double mutant MgrB^−^, RpoS^−^ on K. pneumoniae survival. (B) The contribution of the sensor kinase PhoQ in the environmental survival of K. pneumoniae. (C) MgrB^−^-dependent enhanced survival is dependent upon both RpoS and PhoQ. The WT and MgrB^−^ data are repeated in each panel. Shown is the mean and SEM of six independent assays (in duplicate). Statistical differences were calculated using Kruskal-Wallis tests with Dunn’s test of multiple comparisons across strains at each time point. *, *P < *0.05; **, *P < *0.01; ***, *P < *0.001; ****, *P < *0.0001; n.s., not significant.

MgrB is known to have regulatory function in two key molecular pathways, namely, the repression of PhoPQ and sequestration of the small protein IraM. IraM is an antiadaptor of SprE/RssB, the protein that chaperones the stress response master regulator RpoS to the ClpXP protease for degradation (43). In *mgrB* deletion mutants of Escherichia coli, there is stabilization and subsequent accumulation of RpoS as IraM is no longer associated with MgrB and able to sequester SprE/RssB (44). IraM expression is also regulated positively by PhoPQ (45). We observed an accumulation of RpoS in the MgrB^−^ strain (see Fig. S5 in the supplemental material) and hypothesized that an increase in RpoS, through IraM as its expression and availability is increased, would contribute toward MgrB^−^ K. pneumoniae survival under the stress-induced conditions of solid surface starvation. An *rpoS* deletion mutant (RpoS^−^, AZ139) (Table S1) survived slightly poorly compared with the WT strain (Fig. 5A), suggesting that the RpoS response is required for survival under starvation conditions. However, a *mgrB*, *rpoS* double-deletion mutant strain (MgrB^−^/RpoS^−^, AZ138) (Table S1) enhances survivability only partially compared with the RpoS^−^ strain or the WT strain. Our data would suggest that although RpoS generally promotes environmental survival, there is an additional mechanism(s) that impacts K. pneumoniae environmental survival (Fig. 5A) and that the RpoS effect might be independent of MgrB. Next, we determined the contribution of PhoPQ to the environmental survival of K. pneumoniae. A *phoQ* deletion mutant (PhoQ^−^, AZ107) (Table S1) had lower CFUs than the parental WT strain, especially at day 1 (Fig. 5B). Additionally, a double mutant that inactivated *mgrB* and *phoQ* (MgrB^−^/PhoQ^−^, AZ150) (Table S1) behaved as the parental WT isolate, indicating that PhoQ overactivation in the absence of MgrB is a significant contributor to solid surface starvation survival of K. pneumoniae. Furthermore, we also determined whether a *rpoS* and *phoQ* double-deletion mutant (RpoS^−^/PhoQ^−^, AZ151) (Table S1) would have an additive effect on reduced survivability, with and without *mgrB* inactivation (MgrB^−^/RpoS^−^/PhoQ^−^, AZ155) (Table S1). The double mutant (RpoS^−^/PhoQ^−^) does not have reduced survivability compared with the individual mutants (Fig. 5C), which is indicative of an epistatic interaction. Moreover, the triple mutant (MgrB^−^/RpoS^−^/PhoQ^−^) behaves similarly to the double mutant (RpoS^−^/PhoQ^−^), with the MgrB inactivation unable to rescue the defect, suggesting that the increase in environmental survival through MgrB inactivation is dependent on its dysregulation of PhoPQ and RpoS.

10.1128/mbio.03595-21.5 FIG S5 Western blot analysis of RpoS levels in stationary-phase cultures of WT, MgrB^−^, and MgrB^+^ grown in cation-adjusted Mueller Hinton broth. Shown is an analysis of three independent experiments using ImageJ and a representative blot. Mean with SEM is shown. The RNA polymerase β′ subunit was probed as a loading control. Download FIG S5, PDF file, 0.05 MB. Copyright © 2022 Bray et al. 2022 Bray et al. https://creativecommons.org/licenses/by/4.0/ This content is distributed under the terms of the Creative Commons Attribution 4.0 International license.

## DISCUSSION

This study provides a comprehensive understanding of MgrB-dependent colistin resistance as it pertains to the ability of K. pneumoniae to colonize, survive, and transmit from host to host. We show that the loss of functional MgrB plays a role throughout the pathogenic life cycle of K. pneumoniae. The dysregulation caused by inactive MgrB attenuates K. pneumoniae gut colonization but increases survival outside the host which results in more rapid transmission. This new understanding of the effects of colistin resistance on infection and transmission of K. pneumoniae may guide future investigations into how other antibiotic resistances impact bacteria, as well as re-examine the quarantine and containment practices of patients infected with such pathogens.

While animal models have allowed for the study of K. pneumoniae lung colonization (46, 47), the initial colonization of the gut by K. pneumoniae is poorly understood. Historically, this gap in knowledge is due to ineffective *in vivo* models that utilized antibiotic treatment and infection via oral gavage to establish colonization (48, 49). Using our recently established murine model (35), we assessed the ability of colistin-resistant K. pneumoniae to establish itself in healthy, immunocompetent mice with intact microbiota. Our data show that colistin-resistant K. pneumoniae (MgrB^−^) has a fitness defect in GI colonization (Fig. 2A). While this result is promising for the implications of reduced asymptomatic carriage and shedding, we also observed an increase in survival against lysozyme (Fig. 1A) and recovery of shedding and colonization to WT levels under antibiotic treatment (Fig. 2D). Moreover, we examined several known virulence phenotypes of K. pneumoniae to identify the underlying mechanism for the observed fitness defect in gut colonization by the MgrB^−^ strain. We focused on biofilm formation, hypermucoviscosity, and capsule production, as they are energy-dependent processes and were implicated previously with an antibiotic-resistant-dependent biological cost (33). We observed reduced capsule production by the MgrB^−^ strain that correlates with an increased mucin association (Fig. 3), indicating that the observed fitness defect in gut colonization could be due to an increase in mucosal clearance of MgrB^−^ K. pneumoniae. Furthermore, the colonization defect of MgrB^−^ is alleviated under antibiotic stress (Fig. 2D), suggesting that the colonization resistance by the resident gut microbiota might also contribute toward the colonization defect of MgrB^−^. These results indicate that the defect of MgrB^−^ in gut colonization is complex and multifactorial, with reduced capsule amount and gut microbiota correlating in our study with a defect in gut colonization.

Our model also allowed us to study another poorly understood facet of K. pneumoniae infection, namely, host-to-host transmission (35). K. pneumoniae transmits readily through the fecal-oral route and in a hospital setting has been associated with several superspreading events (36) primarily through fomites (41), making it difficult to track outbreaks. Moreover, Klebsiella species can survive on various surfaces for extensive periods of time, which allows them ample opportunity to be acquired by another susceptible individual (41, 50). Conversely, the environmental survivability of specific K. pneumoniae mutants is greatly understudied. Here, we show that the MgrB^−^ strain surprisingly had enhanced survivability compared with the WT (Fig. 4A) and that this phenotype was not strain specific (Fig. 4B). With the majority of colistin-resistant strains of K. pneumoniae linked to inactivation of the small regulatory protein MgrB (20, 21), it seems that this particular genetic variation would not contribute significantly to environmental survival. However, in addition to regulating PhoPQ, which has a myriad of effects on LPSs and the outer membrane (17, 24–26, 51, 52), MgrB also sequesters IraM, which functions as an antiadaptor to the chaperone protein SprE that binds and delivers the stress response master regulator RpoS for proteolytic degradation (17, 23, 44). Additionally, phosphorylated PhoP upregulates the transcription of IraP, another antiadaptor of SprE (53). Therefore, the consequences of inactivation of MgrB reach beyond colistin resistance and can result in outer membrane alteration and a constitutive stress response from the bacteria. Both responses potentially contribute to the environmental survival and subsequent transmission of K. pneumoniae.

We initially hypothesized that the observed increase in survival would be due to a priming effect as a result of accumulating RpoS. Surprisingly, even though RpoS levels were higher in our MgrB^−^ K. pneumoniae strain, this increase led only to a minor contribution in environmental survival (Fig. 5A), and instead the effect is attributable mainly to the PhoPQ two-component system (Fig. 5B). It is likely that the lipid A modifications observed in MgrB-inactivated K. pneumoniae linked to colistin resistance are also responsible for the increased environmental survival, as lipid A modifications have been linked previously to membrane stabilization and increased survival (54, 55). While MDR is associated with a biological cost that can manifest itself in colonization and affect transmissibility in a hospital setting (fitness cost) (29), studies ascertaining a biological cost associated with antibiotic resistance generally do not focus on host-to-host transmission. Here, we showed that colistin-resistant K. pneumoniae under oral antibiotic pressure colonized as well as the parental strain (Fig. 2D) but transmitted at a higher frequency (Fig. 4D-E). These results suggest a gain of function. Furthermore, a recent molecular epidemiological study by Lapp *et al*. suggests that certain colistin-resistant K. pneumoniae isolates of the ST258 lineage have enhanced transmissibility attributed to point mutations in either *mgrB* or *phoQ* that abrogates their function, as well as the disruption of the putative O-antigen glycosyltransferase *kfoC* (56), suggestive of a role of the O-antigen and acquisition of colistin resistance in transmissibility. K. pneumoniae isolates are genetically diverse (57), and both KPPR1S and ST1322 are genetically distinct from ST258 and do not contain *kfoC*. These data would suggest that the ST258 lineage colistin-resistant strains require a loss of *kfoC* for enhanced transmissibility, whereas isolates tested in our study already lack *kfoC* and by acquiring colistin resistance through *mgrB* inactivation are able to transmit more efficiently.

Taken together, our study experimentally advances our understanding of the cost associated with MgrB-dependent colistin resistance and underscores the importance of considering the full life cycle of the pathogen when characterizing the biological cost associated with antibiotic resistance. While MgrB^−^ K. pneumoniae may colonize healthy individuals poorly, those on antibiotic treatment can become robustly colonized and transmit rapidly. Even in non-antibiotic-treated individuals who likely experience poor colonization, there is the concerning potential for increased horizontal gene transfer of antibiotic resistances, a problem that has already been linked to K. pneumoniae and could grow with increased environmental survival and transmission (58, 59). Colistin-resistant isolates can spread even with appropriate infection control practices. Thus, our study underscores the need for an improved antimicrobial stewardship as well as preventative sterilization methods to curb the potential increase in the transmissibility of colistin-resistant K. pneumoniae.

## MATERIALS AND METHODS

### Ethics statement.

This study was conducted according to the guidelines outlined by National Science Foundation animal welfare requirements and the Public Health Service Policy on Humane Care and Use of Laboratory Animals (60). All animal work was done according to the guidelines provided by the American Association for Laboratory Animal Science (AALAS) (61) and with the approval of the Wake Forest Baptist Medical Center Institutional Animal Care and Use Committee (IACUC). The approved protocol number for this project is A20-084.

### Strain construction.

Strains, plasmids, and primers used in this study are listed in Table S1, S2, and S3, respectively, in the supplemental material. The deletion mutant (*ΔmgrB*, AZ132) was constructed as described (62). Briefly, a kanamycin cassette bookended with FLP recombination target (FRT) sites from plasmid pKD4 with 60-bp homology to the upstream and downstream region of *mgrB* was PCR amplified using the high-fidelity Q5 polymerase (New England BioLabs [NEB]). The purified PCR product was then electroporated (1.8 kV, 400 Ω, 25 μF) into the target strain (AZ63) containing the temperature-sensitive plasmid pKD46 (Table S2), containing the λ red recombination genes downstream of an arabinose-inducible promoter. Subsequently, recombination was carried out as described (62). Successful mutants were selected by plating onto kanamycin (25 μg/mL), and pKD46 (Table S2) was removed by growing the plates overnight at 37°C and confirmed with PCR (*mgrB::kanR*, AZ66) (Table S1 and S3). To remove the kanamycin cassette (*ΔmgrB*, AZ132) (Table S1), pFlp3 containing FLP recombinase and a tetracycline resistance cassette (Table S2) was electroporated into AZ66 (Table S1), and successful mutants were selected for tetracycline (10 μg/mL) resistance and kanamycin (25 μg/mL) sensitivity. The cassette removal was confirmed by PCR (Table S3), and the plasmid was removed by growing successful mutants on 5% sucrose plates.

10.1128/mbio.03595-21.7 TABLE S2 List of plasmids used in the study. Download Table S2, DOCX file, 0.01 MB. Copyright © 2022 Bray et al. 2022 Bray et al. https://creativecommons.org/licenses/by/4.0/ This content is distributed under the terms of the Creative Commons Attribution 4.0 International license.

10.1128/mbio.03595-21.8 TABLE S3 List of primers used in the study. Download Table S3, DOCX file, 0.01 MB. Copyright © 2022 Bray et al. 2022 Bray et al. https://creativecommons.org/licenses/by/4.0/ This content is distributed under the terms of the Creative Commons Attribution 4.0 International license.

To construct all other mutants listed in Table S1 in their appropriate genetic background, PCR was carried out using the Q5 polymerase (NEB) with genomic DNA as the template from their respective mutant in MKP103 background (63). Primers are listed in Table S3, with approximately 500-bp homology on either end of the transposon cassette. λ Red mutagenesis was carried out as described above, and mutants were isolated on agar plates containing chloramphenicol (50 μg/mL). Single colonies were purified and verified through PCR. Removal of the chloramphenicol cassette was done as described previously using the plasmid pCre2 (63). The chromosomal complement of *ΔmgrB* (*mgrB^+^*, AZ141) (Table S1) was constructed as described previously (64) with slight modification. The *mgrB* gene and ∼500 bp upstream and downstream were amplified using WT (AZ55) (Table S1) genomic DNA as the template. Plasmid pKAS46 (Table S2) was digested with NotI and NheI, and the *mgrB* PCR product was cloned using the NEBuilder HiFi DNA assembly kit (NEB), followed by transformation into the Escherichia coli strain S17-1 λpir. Conjugation with *ΔmgrB* (AZ132) (Table S1) and subsequent selection of the complemented strain were carried out as described previously (64).

### Mouse infections and bacterial shedding.

C57BL/6J mice were bred and maintained in the animal facility at Biotech Place, Wake Forest Baptist Medical Center. Specific-pathogen-free (SPF) mice were obtained from Jackson Laboratory (Bar Harbor, ME). Mice were infected as described (35). Briefly, 5- to 7-week-old mice had food and water removed for 4 hours and orally fed two 50-μL doses an hour apart of ∼10^6^ CFU/100 μL of K. pneumoniae suspended in phosphate-buffered saline (PBS) + 2% sucrose from a pipette tip.

Fecal collection and quantification of bacterial shedding were done as described previously (35). To enumerate the bacterial shedding, fecal pellets were homogenized in PBS and serially diluted and plated onto selective antibiotic plates. To induce the supershedder phenotype, mice were infected as described above, and 2 days postinfection, they were administered a single dose of streptomycin (5 mg/200 μL in sterile PBS) via oral gavage. K. pneumoniae was enumerated from fecal shedding for an additional 5 days.

Infections and fecal shedding collection for competition studies (competition index [CI]) were conducted as described above with the infection dose containing a 1:1 ratio of MgrB^−^ (AZ132) (Table S1) and an apramycin resistant derivative of KPPR1 (AZ94) (Table S1) (35). For the enumeration of the colonization in the GI tract, the cecum, ileum, and colon were removed from mice following CO_2_ (2 liters/minute for 5 minutes) euthanasia and subsequent cardiac puncture. The organs were weighed and placed in screw-cap tubes (Fisherbrand; 02-682-558) with 2 to 3 glass beads (BioSpec Products; 11079127). The organs were diluted 1:10 (weight to volume) in PBS, homogenized on a bead-mill (Fisher Brand, Bead Mill 24), and plated as described above. The CI was calculated using the following formula:

$${\text{log}}_{10}\text{ CI}=\frac{\text{mutant output/WT output}}{\text{mutant input/WT input}}$$

For the transmission studies, 5 mice were separated into individual housing and their water was supplemented with 200 mg/L of streptomycin, which they remained on for the duration of the experiment. Twenty-four hours after antibiotic exposure, a single mouse was infected orally with either the WT, MgrB^+^, or MgrB^−^ strain and designated the index mouse. Fecal shedding from the infected index mouse was collected the following day, and colonies were enumerated to ensure colonization. Forty-eight hours after infection of the index mouse, 4 uninfected (contact), individually housed mice were placed in the index mouse’s cage one at a time for 1 hour, after which they were returned to their individual cages. Feces was collected for bacterial enumeration from all 5 mice the next day before repeating the exposure. Contact mice were exposed to the index mouse 5 times.

### Growth curves and generation time.

Selected K. pneumoniae strains were grown overnight at 37°C to stationary phase in LB-Lennox broth (Fisher Scientific; BP1427-2) with constant agitation. The strains were diluted 1:100 in fresh media, and a sample of the subculture was serially diluted and plated onto antibiotic plates for enumeration. The culture was then grown at 37°C with constant agitation with samples collected every hour for 8 hours, serially diluted, and plated for enumeration. The competitive growth experiment was conducted as described above, except both overnight strains were diluted 1:100 in the same culture tube and serial dilutions were plated onto selective antibiotic plates for each strain. The competitive index was calculated as described above. For determination of generation time, strains were grown as described above until the samples reached an optical density at 600 nm (OD_600_) of 0.2; a sample was removed every 15 minutes for up to 75 minutes, diluted, and plated for enumeration. The generation time was calculated using the formula *G* = *t*/*n*, where *G* is the generation time, *t* is the time interval, and *n* is the number of generations as calculated by the formula *n* = 3.3log(*b*/*B*) where *b* is the amount of bacteria at the end of a time interval and *B* is the amount at the beginning of the time interval. Growth experiments in minimal media (M63) were performed as above.

### Lipid A analysis.

Lipid A analysis was conducted using the methods described in Sorensen *et al*. (65). Briefly, pelleted bacteria were smeared onto a MALDI plate with a sterile inoculation loop. A total of 1 μL of buffer, consisting of 0.2 M anhydrous citric acid and 0.1 M trisodium citrate dihydrate, was spotted atop the spotted colony. The plate was then incubated at 110°C for 30 minutes to extract membrane lipids. The plate was then rinsed with endotoxin-free water to wash cell debris. One microliter of 10 mg/mL norharmane matrix suspended in 2:1 chloroform-methanol was spotted onto extracted sample on the MALDI plate. Mass spectra was collected using a Bruker Microflex LRF MALDI-TOF MS instrument in negative ion and reflectron mode.

### Fatty acid analysis.

The bacterial cell pellet was incubated at 70°C for 1 hour in 500 μL of 90% phenol and 500 μL of water. Samples were then cooled on ice for 5 minutes and centrifuged at 9,600 × *g* for 10 minutes. The aqueous layer was collected and 500 μL of water was added to the lower (organic) layer and incubated again. This process was repeated twice, and all aqueous layers were pooled. Two milliliters of ethyl ether was added to the harvested aqueous layers, and this mixture was then vortexed and centrifuged at 900 × *g* for 5 minutes. The organic phase was then collected, and 2 mL of ether was added back to the remaining aqueous phase. This process was carried out twice more. The collected organic layer was then frozen and lyophilized overnight. LPS fatty acids were converted to fatty methyl esters, in the presence of 10 μg pentadecanoic acid (Sigma-Aldrich) as an internal standard, with 2 M methanolic HCl (Sigma-Aldrich) at 90°C for 18 hours. The resultant fatty acid methyl esters were analyzed and quantitated by gas chromatography-mass spectrometry (GC-MS) as follows.

GC-MS separation of derivatives was carried out on a Shimadzu GC-MS 2010 instrument with split injection with an injection temperature of 280°C. The initial oven temperature was 80°C and held for 1 minute. The temperature was increased 25°C/minute until the oven reached 160°C and held for 1 minute. The temperature was increased 10°C/minute until 265°C and then increased 1°C/minute until 270°C. The temperature was held at 270°C for 1 minute followed by a 10°C/minute increase to 295°C where it was maintained for 1 minute. The total run time per sample was 25.2 minutes. The column head pressure was 100 kPa with a mass selective detector (MSD). Transfer line temperature was 180°C and mass range was *m/z* 50 to 400. The identification of metabolites was performed using the standard National Institute of Standards and Technology (NIST) 08 standard and Golm Metabolome Database (GMD) mass spectra libraries and by comparison with authentic standards. Percent fatty acid composition was calculated by dividing the concentration of each individual fatty acid by the total concentration for all fatty acids observed in the fatty acid analysis for each strain. Data processing was performed using a pipeline in KNIME.

### Solid surface survival.

To determine the bacterial survivability, strains of interest were grown overnight at 37°C with constant agitation. The overnight cultures were centrifuged at 21,000 × *g* for 25 minutes, washed twice with 1× PBS, and resuspended to final adjusted OD_600_ of 4.0. Twenty-microliter aliquots of the bacterial suspensions were spotted onto nitrocellulose membranes (MF-Millipore; HAWP02500) on 2-mL pads of 1% agarose (Fisher; BP160-500) in 6-well polystyrene plates (Costar; 3516) and air dried. At designated time intervals, the membrane was removed, placed in 1 mL PBS, and vortexed to dislodge the bacteria, and the suspension was serially diluted and plated onto LB agar plates (Fisher; BP9745-2) In between sampling, the plates were sealed with parafilm (Bemis; PM-999) and placed in the dark at room temperature.

### Biofilm quantification.

For biofilm assays, overnight cultures were grown as described above in LB-Lennox broth and diluted 1:100 in M63 media with 0.5% glucose. One-hundred microliters of the bacterial suspension was placed in the wells of a 96-well round-bottom culture plate (Costar), sealed (Fisherbrand Thermal Adhesive Sealing Film), and incubated for 24 hours at 37°C. Staining and quantification were carried out as described (66).

### Mucoviscosity assay.

Mucoviscosity of the K. pneumoniae isolates was determined using a low-speed centrifugation (1,000 × *g*, 5 min) of bacterial cultures adjusted to 1 OD_600_ and measuring the OD_600_ of the supernatant as described previously (67).

### Uronic acid measurement.

To determine the amount of capsular polysaccharide, uronic acid content was measured as described previously (67).

### Mucin binding assay.

Mucin binding was determined as described previously (39, 68). Nunc Polysorp plates (Thermo Fisher Scientific; 475094) were coated with 100 μL of 50 μg semipurified Millipore mucin (catalog [cat.] number 499643-500MG) by centrifugation (250 × *g*, 3 minutes) and incubated overnight at 37°C. The bound mucin was washed 3 times, and then ∼10^5^ CFU of K. pneumoniae in 100 μL was added. The plate was centrifuged (250 × *g*, 3 minutes) and incubated at 37°C for 60 min. The wells were washed 15 times to remove any unbound bacteria. Subsequently, the wells were treated with 200 μL of PBS-0.5% Triton X-100 (Sigma-Aldrich) for 30 minutes followed by vigorous mixing. Samples were then plated in triplicate from the wells for enumeration.

### Antimicrobial resistance assays.

The polymyxin and lysozyme resistance assays were carried out as described (69, 70). For the lysozyme assay, K. pneumoniae strains were grown overnight then diluted 1:100 in fresh media and grown to mid-log phase (OD_600_ of 0.4 to 0.5), centrifuged (21,000 × *g*, 20 min), and washed with PBS twice before being diluted 1:100 in Tris-EDTA (TE) buffer with 1% tryptic soy broth (TSB) (BD). Next, 96-well plates were prepared with 100 μL of appropriate concentrations of chicken egg lysozyme suspended in TE + 1% TSB, and 100 μL of the bacterial suspension was added to wells in triplicate. The plate was incubated at 37°C for 1 hour before samples were taken from each well, diluted, and plated for enumeration.

### Gel electrophoresis and analysis.

Western blots were carried out as described previously (71) with modifications. Bacterial cultures were grown overnight in cation-adjusted Mueller-Hinton Broth (caMHB), diluted 1:100 in fresh caMHB, and grown for 16 hours to stationary phase. A total of 500 μL of the bacterial culture was pelleted (21,000 × *g*, 25 minutes) and resuspended in a volume of 2× Laemmli sample buffer (Bio-Rad) equal to sample OD_600_/6 × 250. Samples were boiled at 100°C for 20 minutes. Fifteen microliters of each sample was loaded into the wells of a 12% polyacrylamide gel (Bio-Rad; 4561045) and run at 150 V for 75 minutes. Transfer from the gel to a nitrocellulose membrane (Bio-Rad; 1704158) was carried out using the Bio-Rad Trans-Blot Turbo transfer system. The membrane was probed with anti-RNA sigma S antibody (BioLegend; cat. number 663703) and anti-E. coli RNA Polymerase β Prime Antibody (BioLegend; cat. number 662904) as a loading control. The secondary antibody was goat anti-mouse IgG (H+L)-horseradish peroxidase (HRP) conjugate (Bio-Rad; cat. number 1706516). The blot was developed with Clarity western ECL substrate (Bio-Rad; 170-5061). Final imaging was done using the Bio-Rad ChemiDoc MP imaging system, and blot analysis was conducted using ImageJ (72).

### Statistical analysis.

All statistical analyses were performed using Prism 9.0 (GraphPad Software, Inc., San Diego, CA). Unless otherwise specified, differences were determined using the Mann-Whitney *U* test (comparing two groups), the Kruskal-Wallis test with Dunn’s postanalysis (comparing multiple groups), the Wilcoxon signed-rank test, or Fisher’s exact test.
