# Supplementary figures and images for: MgrB-Dependent Colistin Resistance in Klebsiella pneumoniae Is Associated with an Increase in Host-to-Host Transmission
Source: mBio. 2022 Mar 21;13(2):e03595-21. doi: 10.1128/mbio.03595-21 (PMC9040857; doi:10.1128/mbio.03595-21)

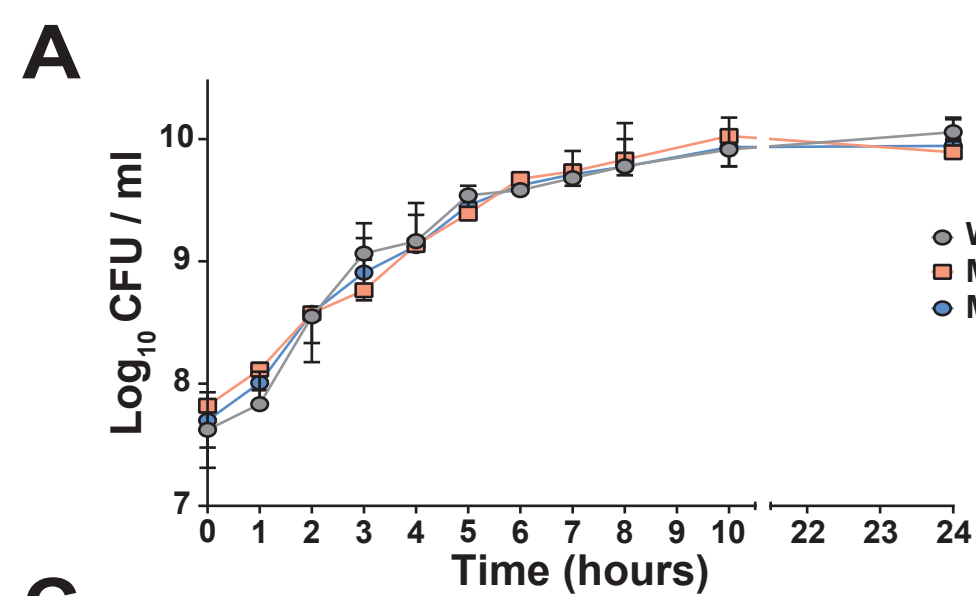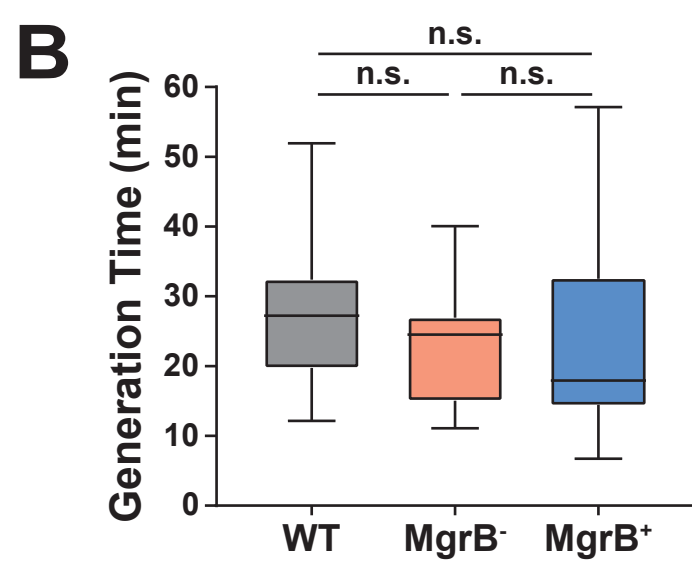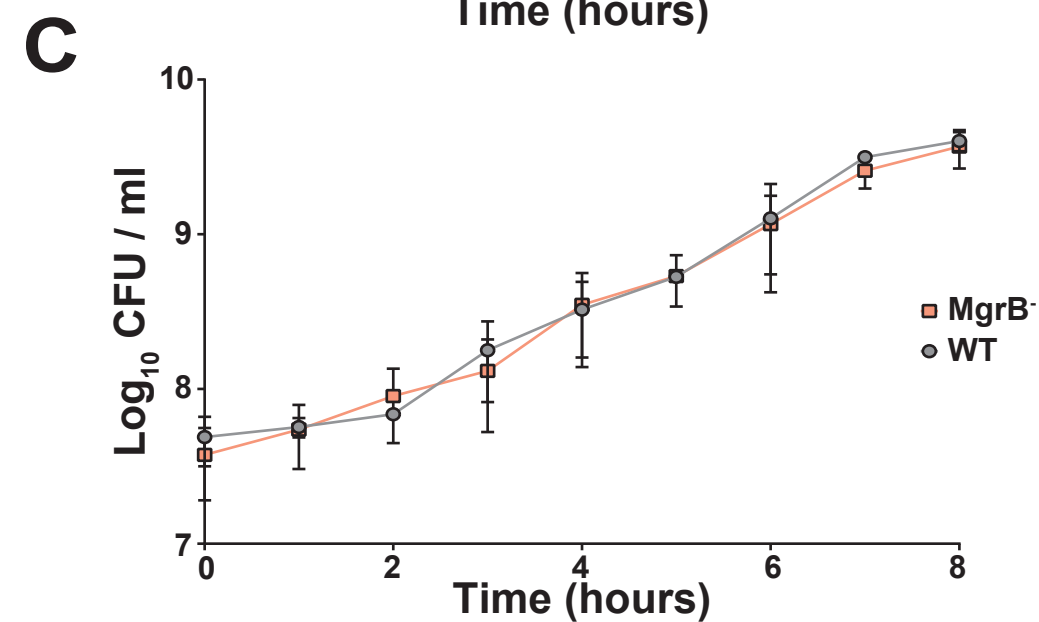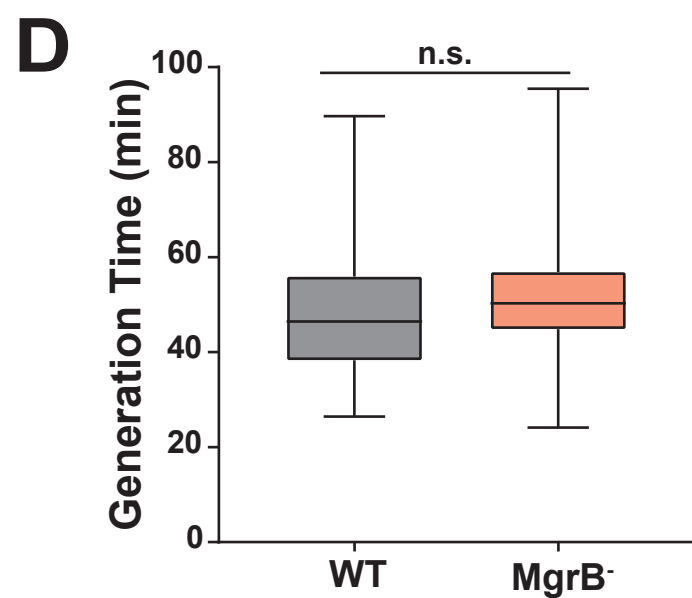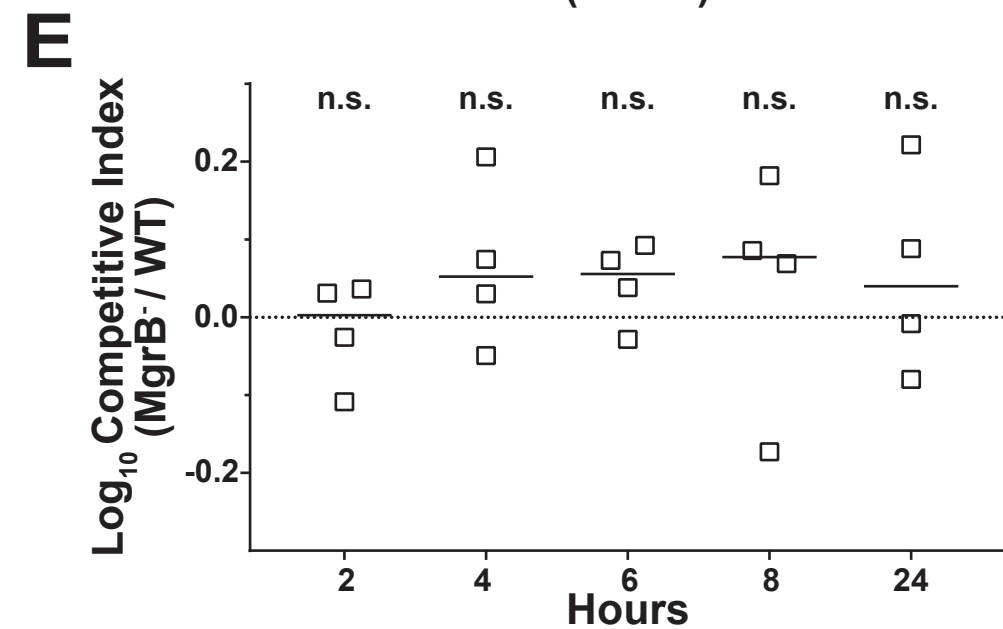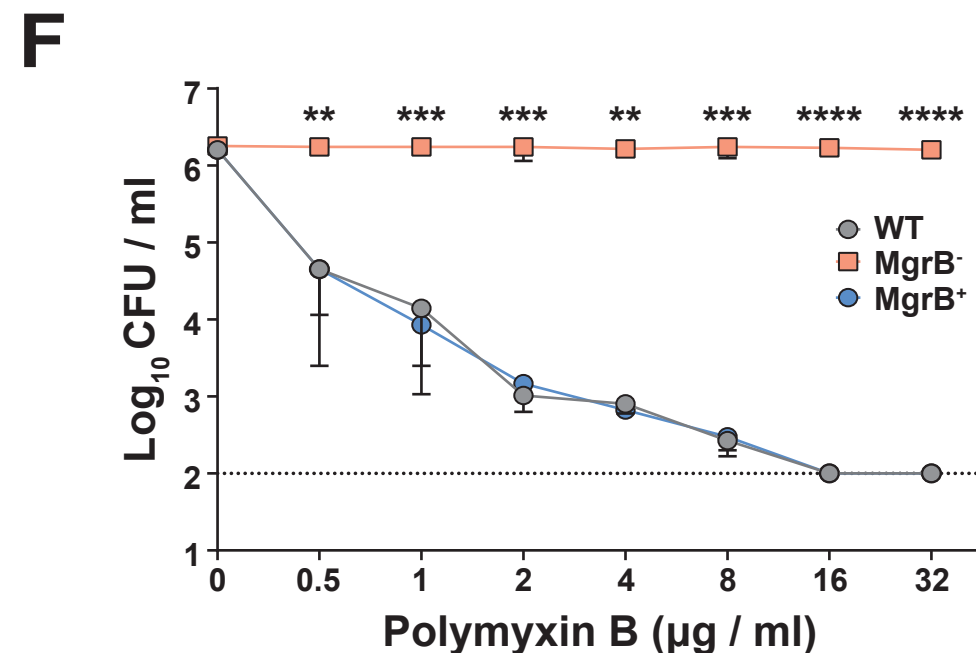

Supplement: FIG S1 [file mbio.03595-21-sf001.pdf]

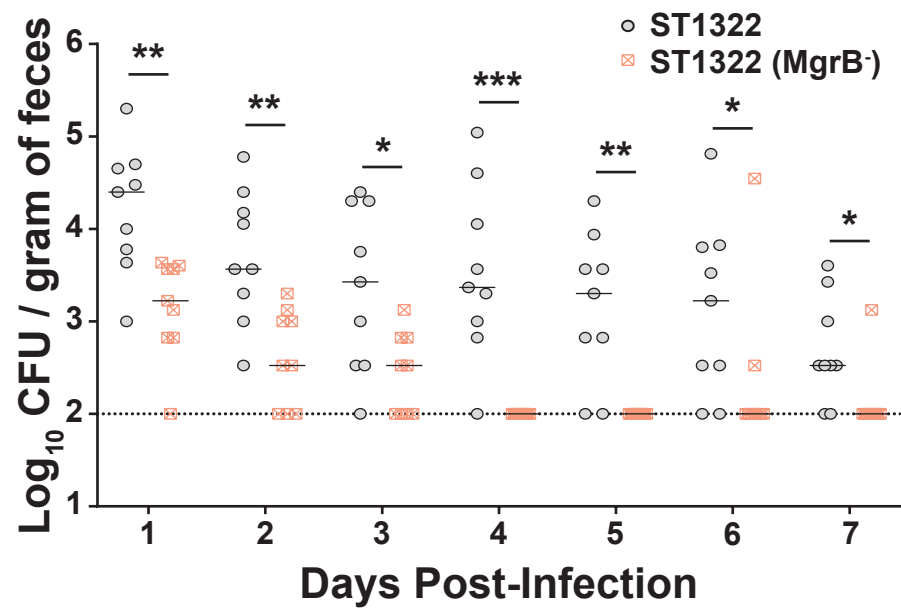

Supplement: FIG S2 [file mbio.03595-21-sf002.pdf]

**A**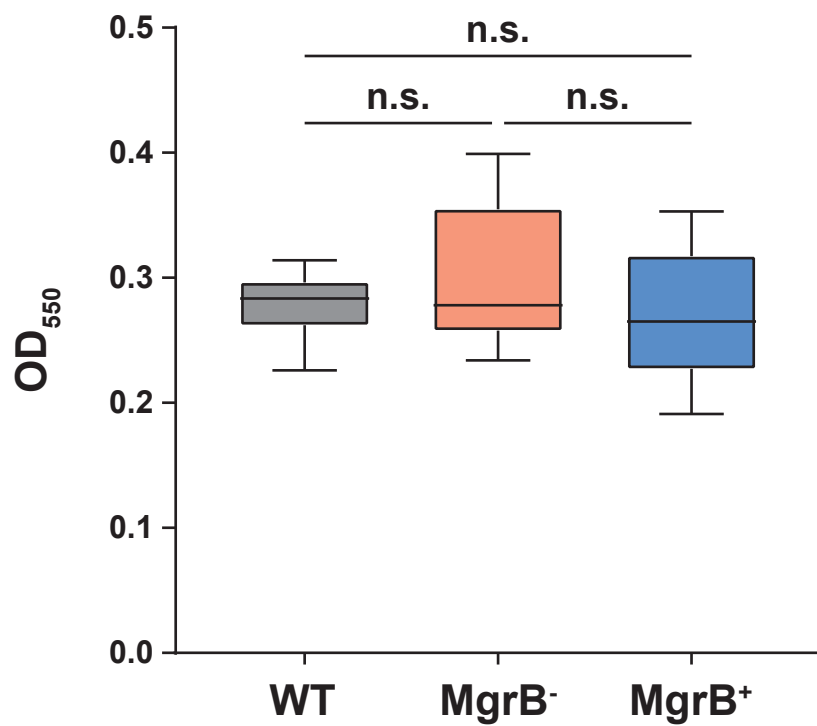**B**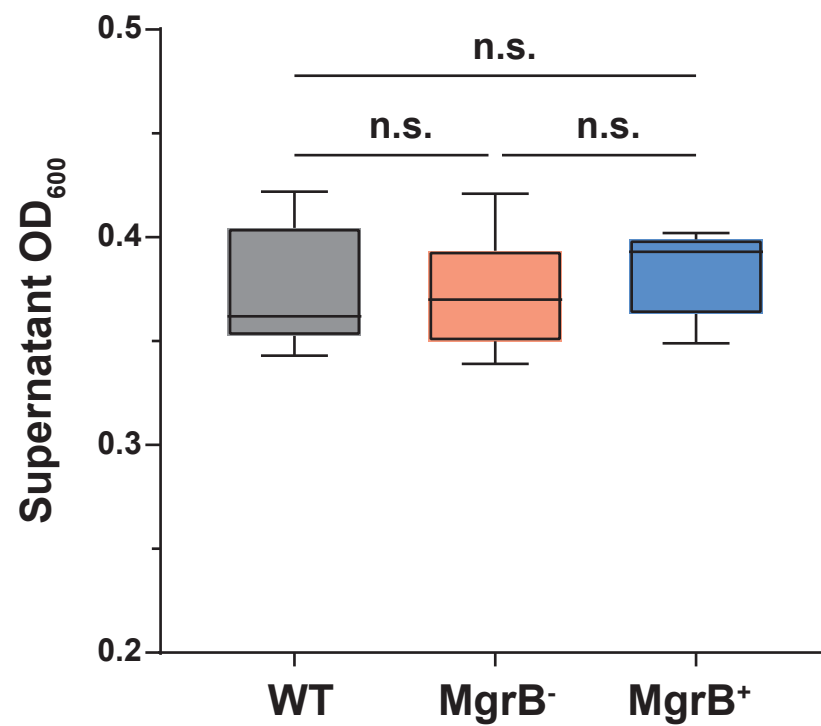

Supplement: FIG S3 [file mbio.03595-21-sf003.pdf]

**A**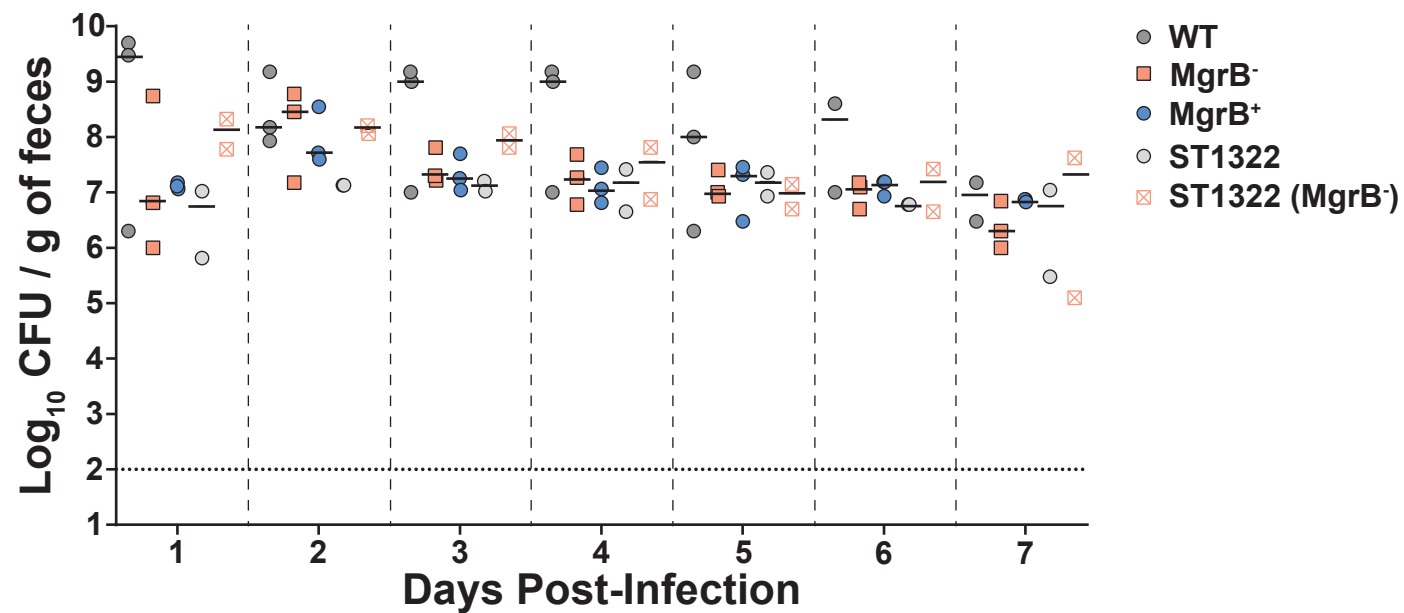**B**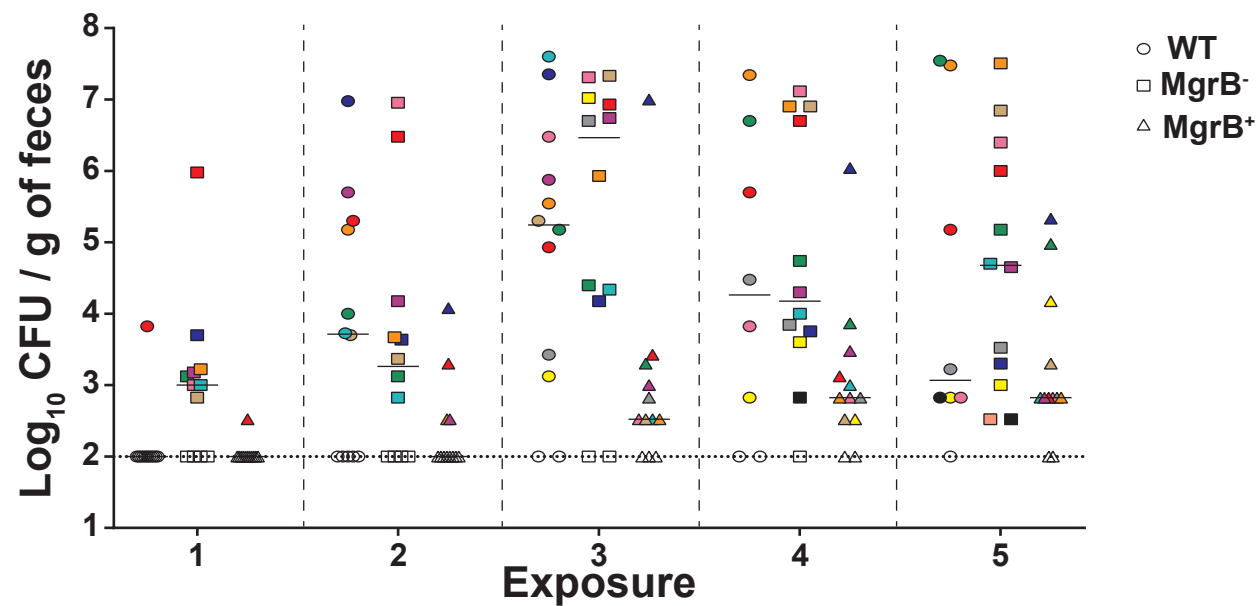**C**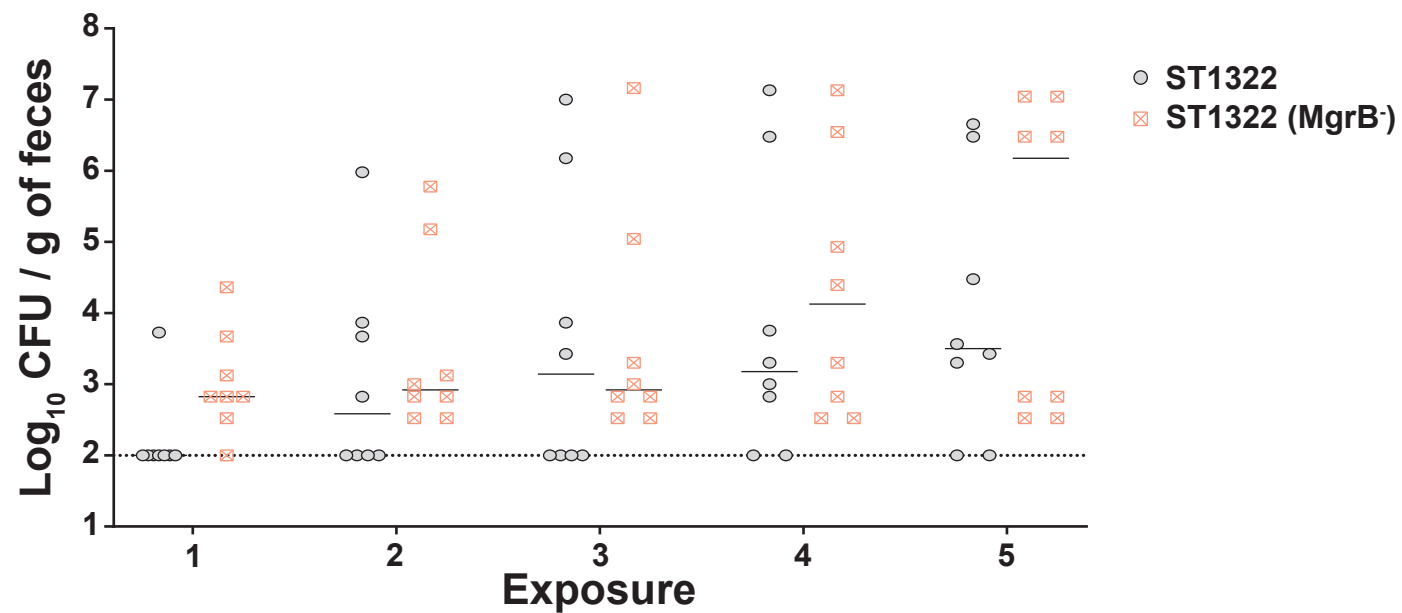

Supplement: FIG S4 [file mbio.03595-21-sf004.pdf]

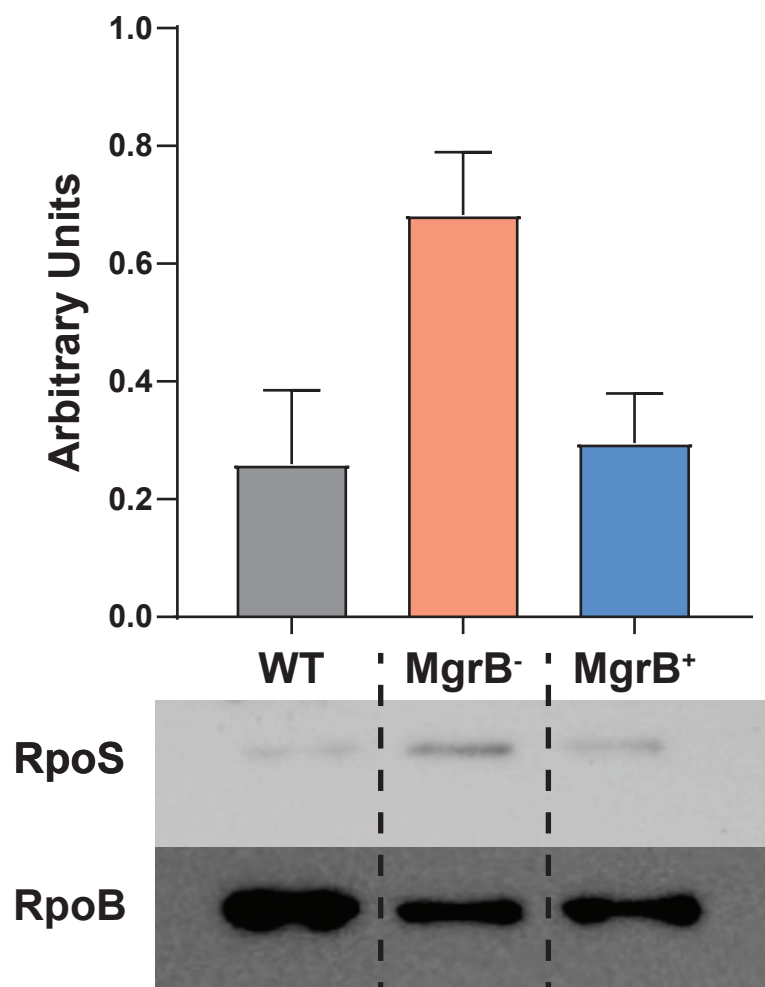

Supplement: FIG S5 [file mbio.03595-21-sf005.pdf]
