# Supplementary material for: MgrB-Dependent Colistin Resistance in Klebsiella pneumoniae Is Associated with an Increase in Host-to-Host Transmission
Source: mBio. 2022 Mar 21;13(2):e03595-21. doi: 10.1128/mbio.03595-21 (PMC9040857; doi:10.1128/mbio.03595-21)
Supplement: TABLE S1 [file mbio.03595-21-st001.docx]

**Table S1.**

| **Strain** | **Genotype** | **Description** | **Reference or source** |
| --- | --- | --- | --- |
| AZ55 | KPPR1S (ATCC43816), *rif^r^, str^r^* | *str^r^* derivative of KPPR1 | (1) |
| AZ66 | KPPR1S *mgrB::kan, rif^r,^, str^r^, kan^r^* | *mgrB::kan* mutant of AZ55 | This study |
| AZ94 | KPPR1 (ATCC43816),  *rif^r,^, apra^r^* | Derivative of KPPR1 with apramycin resistance cassette at *att::*Tn7 site | (2) |
| AZ107 | KPPR1S *phoQ::cam,*  *rif^r^, str^r^, cam^r^* | *phoQ::cam* from AZ176 into AZ55; plasmid cured | This study |
| AZ124 | KPPR1S *ΔwcaJ,*  *rif^r^, str^r^* | Δ*wcaJ* (CPS^-^) derivative of AZ55 | (3) |
| AZ132 | KPPR1S *ΔmgrB,*  *rif^r^,* *str^r^* | *ΔmgrB*, clean deletion created from AZ66 using pFlp3; plasmid cured | This study |
| AZ136 | MKP103 *rpoS::cam,*  *cam^r^* | Arrayed library MKP103 with transposon element in *rpoS::cam,* (strain: tnkp1_lr150214p07q164) | (4) |
| AZ138 | KPPR1S *ΔmgrB, rpoS::cam*  *rif^r^, str ^r^, cam^r^* | *rpoS::cam* from AZ136 into AZ132; plasmid cured | This study |
| AZ139 | KPPR1S *rpoS::cam, rif^r^, str^r^, cam^r^* | *rpoS::cam* from AZ136 into AZ55; plasmid cured | This study |
| AZ141 | KPPR1S *mgrB,^+^*  *rif^r^, str^r^* | *mgrB^+^* (chromosomally complemented) derivative of AZ132 | This study |
| AZ150 | KPPR1S *ΔmgrB, phoQ::cam, rif^r^, str^r^, cam^r^* | *phoQ::cam* from AZ107 into AZ132; plasmid cured | This study |
| AZ151 | KPPR1S *ΔrpoS, phoQ::cam, rif^r^, str^r^, cam^r^* | *phoQ::cam* from AZ107 into AZ142; plasmid cured | This study |
| AZ63 | KPPR1S *+ pKD46, rif^r^, str^r^, spec^r^* | AZ55 with pKD46 plasmid for lambda red recombination | This study |
| AZ142 | KPPR1S *ΔrpoS,* *rif^r^*, *str^r^* | *ΔrpoS*, clean deletion created from AZ139 using pCre2; plasmid cured | This study |
| AZ143 | KPPR1S *ΔrpoS + pKD46,* *rif^r^, str^r^, spec^r^* | AZ142 with pKD46 for lambda red recombination | This study |
| AZ137 | KPPR1S *ΔmgrB,* *+pKD46,*  *spec^r^* | AZ132 with pKD46 for lambda red recombination | This study |
| AZ140 | S17-1 λpir + *pKAS46(mgrB^+^) kan^r^* | S17-1 λpir with pKAS46(*mgrB^+^)* for chromosomal complementation | This study |
| AZ16 | S17-1λpir  *recA thi pro hsdR- M+ RP4::2-Tc::Mu::Km Tn7 tp^r^ str^r^ λpir* | S17-1 λpir *E. coli*  Donor strain for conjugation | (5) |
| AZ40 | SM10(λpir)  *thi-J thr leu tonA lacY supE recA::RP4-2-Tc::Mu, kan^r^, λpir* | SM10(λpir) *E. coli*  Donor strain for conjugation | (5) |
| AZ152 | KPPR1S *ΔrpoS, phoQ::cam + pKD46, rif^r^, str^r^, spec^r^* | AZ151 with pKD46 plasmid for lambda red recombination | This study |
| AZ155 | *KPPR1S ΔrpoS, phoQ::cam, mgrB::kan, rif^r,^, str^r^, kan^r^, cam^r^* | *mgrB::kan* from AZ66 into AZ151; plasmid cured | This study |
| AZ176 | MKP103, *phoQ::cam, cam^r^* | Arrayed library MKP103 with transposon element in *phoQ::cam* (strain: tnkp1_lr150117p19q179) | (4) |
| AZ99 | ST1322, str^r^ | ST1322 strain, fecal isolate made Str^r^ | (6, 7) |
| AZ178 | ST1322 *+ pKD46, str^r^, spec^r^* | AZ99 with pKD46 for lambda red recombination | This study |
| AZ179 | ST1322, *mgrB::kan, str^r^, kan^r^* | *mgrB::kan* from AZ66 into AZ178; plasmid cured | This study |

*rif^r^*, rifampicin resistant; *str^r^*, streptomycin resistant; *apra^r^*; apramycin resistant, *cam^r^*, chloramphenicol resistant; *spec^r^*, spectinomycin resistant; *kan^r^*, kanamycin resistant

1. Palacios M, Broberg CA, Walker KA, Miller VL, D'Orazio SEF. 2017. A Serendipitous Mutation Reveals the Severe Virulence Defect of a Klebsiella pneumoniae fepB Mutant. mSphere 2:e00341-17.

2. Agard MJ, Ozer EA, Morris AR, Piseaux R, Hauser AR, Ehrt S. 2019. A Genomic Approach To Identify Klebsiella pneumoniae and Acinetobacter baumannii Strains with Enhanced Competitive Fitness in the Lungs during Multistrain Pneumonia. Infection and Immunity 87:e00871-18.

3. Walker KA, Miner TA, Palacios M, Trzilova D, Frederick DR, Broberg CA, Sepúlveda VE, Quinn JD, Miller VL, Goldberg JB. 2019. A Klebsiella pneumoniae Regulatory Mutant Has Reduced Capsule Expression but Retains Hypermucoviscosity. mBio 10:e00089-19.

4. Ramage B, Erolin R, Held K, Gasper J, Weiss E, Brittnacher M, Gallagher L, Manoil C. 2017. Comprehensive Arrayed Transposon Mutant Library of Klebsiella pneumoniae Outbreak Strain KPNIH1. J Bacteriol 199.

5. Simon R, Priefer U, Pühler A. 1983. A Broad Host Range Mobilization System for In Vivo Genetic Engineering: Transposon Mutagenesis in Gram Negative Bacteria. Bio/Technology 1:784-791.

6. Martin RM, Cao J, Brisse S, Passet V, Wu W, Zhao L, Malani PN, Rao K, Bachman MA. 2016. Molecular Epidemiology of Colonizing and Infecting Isolates of Klebsiella pneumoniae. mSphere 1.

7. Young TM, Bray AS, Nagpal RK, Caudell DL, Yadav H, Zafar MA. 2020. Animal Model To Study Klebsiella pneumoniae Gastrointestinal Colonization and Host-to-Host Transmission. Infect Immun 88.
