# Supplementary material for: MgrB-Dependent Colistin Resistance in Klebsiella pneumoniae Is Associated with an Increase in Host-to-Host Transmission
Source: mBio. 2022 Mar 21;13(2):e03595-21. doi: 10.1128/mbio.03595-21 (PMC9040857; doi:10.1128/mbio.03595-21)
Supplement: TABLE S2 [file mbio.03595-21-st002.docx]

**Table S2.**

| **Plasmid** | **Description** | **Antibiotic marker** | **Source** |
| --- | --- | --- | --- |
| pKD46 | λ red recombinase genes downstream of *araBAD* promoter; temperature sensitive (32°C) | *spec^r^* | (1) |
| pFlp3 | Flp recombinase for removing FRT *kan* cassette | *amp^r^*, *tet^r^* | (2) |
| pKAS46 | Vector for allelic exchange, contains *rpsL* for streptomycin counter-selection | *kan^r^* | (3) |
| pCre2 | Cre recombinase for removing loxP *cam* cassette | *amp^r^* | (4) |
| pKD4 | Kanamycin cassette with FRT sites | *kan^r^* | (5) |

*amp^r^*, ampicillin resistant; *kan^r^*, kanamycin resistant; *spec^r^*, spectinomycin resistant; *tet^r^*, tetracycline resistant

1. Tyler JS, Beeri K, Reynolds JL, Alteri CJ, Skinner KG, Friedman JH, Eaton KA, Friedman DI. 2013. Prophage Induction Is Enhanced and Required for Renal Disease and Lethality in an EHEC Mouse Model. Plos Pathogens 9.

2. Choi KH, Gaynor JB, White KG, Lopez C, Bosio CM, Karkhoff-Schweizer RR, Schweizer HP. 2005. A Tn7-based broad-range bacterial cloning and expression system. Nat Methods 2:443-8.

3. Karen Skorupski RKT. 1995. Positive selection vectors for allelic exchange. Gene doi:10.1016/0378-1119(95)00793-8.

4. Bailey J, Manoil C. 2002. Genome-wide internal tagging of bacterial exported proteins. Nat Biotechnol 20:839-42.

5. Datsenko KA, Wanner BL. 2000. One-step inactivation of chromosomal genes in Escherichia coli K-12 using PCR products. Proc Natl Acad Sci U S A 97:6640-5.
