# Supplementary material for: MgrB-Dependent Colistin Resistance in Klebsiella pneumoniae Is Associated with an Increase in Host-to-Host Transmission
Source: mBio. 2022 Mar 21;13(2):e03595-21. doi: 10.1128/mbio.03595-21 (PMC9040857; doi:10.1128/mbio.03595-21)
Supplement: TABLE S3 [file mbio.03595-21-st003.docx]

**Table S3.**

| **Primer Name** | **Description** | **Orientation** | **Sequence (5’->3’)** |
| --- | --- | --- | --- |
| mgrB-pkd4-US | λ red knockout for mgrB | Forward | TAAGAAGGCCGTGCTATCCTGGCGACATTGCGTACTGATGCGGAGAGTGGAGTGAAAAAATGTGTAGGCTGGAGCTGCTTC |
| mgrB-pkd4-DS | λ red knockout for mgrB | Reverse | TTCATTCTACCACCCGCGGCGCAGAAGGAAATCAGTCGGCAGAAAAATGTCTTACCACGGCATGGGAATTAGCCATGGTCC |
| Kan-Forward K1 | Sequence of Kan marker off pKD4 | Forward | GGGCACAACAGACAATCGGC |
| Kan-Reverse-K2 | Sequence of Kan marker off pKD4 | Reverse | GCAGTTCATTCAGGGCACCG |
| mgrB-US | Upstream of coding sequence of mgrB | Forward | CCTGGCGTGATTTTGACACG |
| mgrB-DS | Downstream of coding sequence of mgrB | Reverse | AGCCAGCGATGCCAGATTT |
| Internal-mgrB-F | Internal mgrB | Forward | TACGGTGGGTTTTACTGATAGTC |
| Internal-mgrB-R | Internal mgrB | Reverse | TTAATAGTGCAAATGCCGCTG |
| PhoQ-F | Upstream of PhoQ | Forward | GGCATATCTTCCCGCTGTCA |
| PhoQ-R | Downstream of PhoQ | Reverse | AGGATGTGCAGATGGCTGTC |
| RpoS-upstream | Upstream of RpoS | Forward | TTGCAGGCAGTAAGGGACAG |
| RpoS- downstream | Downstream of RpoS | Reverse | GAAGTCCTCGATGCCCATGT |
| mgrB-US-NheI | mgrB^+^ into pKas46 | Forward | GCGCTTTAAATTTGCGCATGCTAGCCGTAAGATTTCTGAACAAATG |
| mgrB-DS-NotI | mgrB^+^ into pKas46 | Reverse | CGATGGCGCCAGCTGCAGGCGGCCGCCGGCGAGAATGACTTTATTTAC |
